# Genetic and RNA-related molecular markers of trastuzumab-chemotherapy-associated cardiotoxicity in HER2 positive breast cancer: a systematic review

**DOI:** 10.1186/s12885-022-09437-z

**Published:** 2022-04-12

**Authors:** Mattia Lunardi, Ahmed Al-Habbaa, Mahmoud Abdelshafy, Matthew G. Davey, Ahmed Elkoumy, Sandra Ganly, Hesham Elzomor, Christian Cawley, Faisal Sharif, James Crowley, Michael Kerin, William Wijns, Aoife Lowery, Osama Soliman

**Affiliations:** 1 Discipline of Cardiology, Saolta Group, Galway University Hospital, Health Service Executive and CORRIB Core Lab, National University of Ireland Galway (NUIG), Galway, H91 TK33 Ireland; 2 Division of Cardiology, Department of Medicine, University of Verona, Verona, Italy; 3 The Lambe Institute for Translational Medicine and CURAM, National University of Ireland Galway (NUIG), Galway, Ireland; 4 Department of Cardiology, Faculty of Medicine, Al-Azhar University, Cairo, Egypt; 5 Discipline of Surgery, School of Medicine, Lambe Institute for Translational Research, National University of Ireland Galway, Galway, Ireland; 6 Precision Cardio-Oncology Research Enterprise (P-CORE), National University of Ireland, Galway, Ireland

**Keywords:** Genetic markers, Polymorphisms, miRNA, HER2, Breast cancer, Cardiotoxicity

## Abstract

Cancer-therapy related cardiotoxicity (CTRCT) is a significant and frequent complication of monoclonal antibody directed therapy, especially Trastuzumab, for human epidermal growth factor receptor 2 (HER2) overexpressing breast cancers. Reliable, clinically available molecular predictive markers of CTRCT have not yet been developed. Identifying specific genetic variants and their molecular markers, which make the host susceptible to this complication is key to personalised risk stratification. A systematic review was conducted until April 2021, using the Medline, Embase databases and Google Scholar, to identify studies genetic and RNA-related markers associated with CTRCT in HER2 positive breast cancer patients. So far, researchers have mainly focused on HER2 related polymorphisms, revealing codons 655 and 1170 variants as the most likely SNPs associated with cardiotoxicity, despite some contradictory results. More recently, new potential genetic markers unrelated to the HER2 gene, and linked to known cardiomyopathy genes or to genes regulating cardiomyocytes apoptosis and metabolism, have been detected. Moreover, microRNAs are gaining increasing recognition as additional potential molecular markers in the cardio-oncology field, supported by encouraging preliminary data about their relationship with cardiotoxicity in breast cancers. In this review, we sought to synthesize evidence for genetic variants and RNA-related molecular markers associated with cardiotoxicity in HER2-positive breast cancer.

**Supplementary Information:**

The online version contains supplementary material available at 10.1186/s12885-022-09437-z.

## Introduction

Breast cancer (BC) represents a major medical problem worldwide being the most commonly diagnosed cancer and the leading cause of cancer mortality among females [1].

About 15 to 20% of all breast cancers overexpress Human Epidermal Growth Factor Receptor 2 (HER2) which is recognized as a more aggressive biological subtype, therefore potentially leading to worse outcomes [2].

Adjuvant treatment of patients with HER2 positive BC traditionally comprised systemic therapy with anthracycline containing regimens, with or without radiotherapy and other therapies (i.e. anti-oestrogen therapy). The introduction of HER2 targeted therapy (initially the monoclonal antibody Trastuzumab) has improved cancer survival in patients with HER2 positive BC [3]. However, cardiotoxicity associated with HER2 targeted therapies has become a major concern. In addition, the effect of other concomitant chemotherapy molecules may increase even more the single-drug adverse effect, as demonstrated for the association of Trastuzumab and Doxorubicin, which type II topoisomerase downregulation synergizes the cardiotoxicity [4]. Some clinical risk factors have been identified as predictors of cardiotoxicity, such as hypertension and coronary artery disease, despite non-specific of HER2 targeted therapies effects [5, 6].

It has been reported that cardiomyopathy develops in up to 30% of patients during or following HER2 targeted therapy [7]. Yet, identifying patients at risk and the underlying mechanisms of HER2 targeted therapy induced cardiomyopathy remain undetermined. To date, mostly non-specific clinical risk factors has been.

Candidate gene analyses (i.e. HER2 gene), and genome wide association studies (GWAS) have identified common genetic variants associated with cancer therapy induced cardiomyopathy [8]. In addition, the importance of RNA-related molecular markers, such as microRNAs, is increasingly evident [9]. Their role in physiological and pathological conditions such as cell differentiation, replication and regeneration is well established [10]. However patterns of miRNA expression during chemotherapy induced cardiotoxicity are not yet established [11].

Identifying genetic and molecular markers of chemotherapy induced cardiotoxicity could lead to early identification and improved surveillance of high-risk patients and the timely initiation of cardioprotective drugs [12]. Consequently, it might mitigate the risk of cardiomyopathy and/or the undesirable need to stop or pause HER2 targeted therapies due to cardiotoxicity [13].

The aim of this review is to synthesize and discuss the current evidence for genetic and RNA-related markers associated with cancer therapy-related cardiotoxicity (CTRCT) in HER-2 positive breast cancer patients.

## Material and methods

### Search strategy

This is a systematic review conducted and reported according to the Preferred Reporting Items for Systematic Reviews and Meta-Analyses (PRISMA) guidelines [14]. Potential studies were retrieved based on a literature research of articles published until February 2022, using the Medline, Embase databases and Google Scholar. The full search details are summarized in the Supplementary Table 1. Institutional review board approval was not required for this study. Data sharing is not applicable to this article as no datasets were generated or analysed during the current study.

### PICO criteria

#### Population

Patients with HER2 positive BC.

#### Intervention

Trastuzumab with or without other cancer therapy including chemotherapy and radiotherapy.

#### Comparison

Genetic and molecular markers in patients who developed and those who did not develop Cancer-therapy related cardiotoxicity.

#### Outcome

Cancer therapy related cardiotoxicity (definitions utilized in individual studies are listed in Table 1).

**Table 1:** Summary of CTRCT events and related genetic determinants in studies investigating HER2 SNPs

Author	Investigated SNP (rsID)	No of patients	Genetic variant carriers	CTRCT definition	Total CTRCT events	Case- Events^a^	Control-Events^b^	OR	95%CI	*p* value
Gómez Peña et al. [15]	1,136,201 (HER2 655 A > G)	78	28 (35.6%)	LVEF↓ > 10% resulting < 50%; or LVEF↓ > 15%; or any LVEF↓, resulting < 45%; or CHF	9 (32.1%)	9/28 (32.1%)	6/50 (12%)	3.41	1.02–11.96	0.039
Beauclair et al. [16]	1,136,201 (HER2 655 A > G)	61	25 (41%)	LVEF↓ > 20%	5 (8.2%)	5/25 (20%)	0	NA	NA	0.004
Lemieux et al. [17]	1,136,201 (HER2 655 A > G)	73	21 (29%)	LVEF↓ > 10% resulting < 50%; or any LVEF↓, resulting < 45%;	10 (13.7%)	6/21 (28.6%)	4/52 (7.7%)	4.67	1.01–19.94	0.028
Roca L et al. [2]	1,136,201 (HER2 655 A > G)	132	53 (40%)	Any LVEF↓ resulting < 50%; or LVEF↓ > 15%; or discontinuation of trastuzumab due to CTRCT	13 (9.8%)	9/53 (16.9%)	4/79 (5%)	3.79	1.09–14.01	0.026
Tan et al. [18]	1,136,201 (HER2 655 A > G)	91	27 (29.7%)	LVEF↓ > 10% resulting < 53%; or CHF; or ACS; or fatal arrhythmia	26 (28.6%)	13/27 (48.1%)	13 (20.3%)	7.99	1.79–35.76	0.007
Peddi et al. [19]	1,136,201 (HER2 655 A > G)	662	238 (35.5%)	LVEF↓ > 10%; or CHF	115 (17.3%)	47/238 (19.7%)	68/424 (16%)	NA	NA	0.65
Stanton et al. [20]	1,058,808 (HER2 1170 C > G)	140	111 (79.3%)	LVEF↓ > 15%; or CHF	29 (21%)	19/111 (17.1%)	10/29 (34.5%)	2.60	1.02–6.62	0.004
Boekhout et al. [21]	1,058,808 (HER2 1170 C > G)	206	NA	LVEF↓ > 15%; or LVEF < 45%	36 (17.4%)	NA	NA	0.09	0.02–0.45	0.003

^a^Case-Events: CTRCT events among genetic variant carriers. ^b^Control-Events: CTRCT events among genetic variants non-carriers

*Abbreviations*: *NA* Not available, *CHF* Congestive heart failure, *LVEF* Left ventricle ejection fraction, *ACS* Acute coronary syndrome

### Inclusion and exclusion criteria

Adult (≥18 years) HER2 overexpressing breast cancer patients undergoing chemotherapy and adjuvant targeted monoclonal antibody therapy against HER2 were included. We included all clinical studies (randomized controlled trials (RCTs), prospective or retrospective observational studies) investigating the association between genetic and RNA-related molecular determinants (i.e. single-nucleotide polymorphisms (SNPs), non-coding RNA, microRNA) and cardiac adverse events regardless of its definition. Case reports, studies not reporting cardiotoxicity data, reviews, and articles not in the English language were excluded.

### Study selection

Two researchers (M.L. and A.A.) independently reviewed abstracts and full texts in a blinded standardized manner. Furthermore, references in selected articles were independently cross-checked by the 2 researchers for other relevant studies. Disagreements between the researchers to include a study were discussed and resolved by senior contributors before final approval.

### Data extraction

Two authors (M.L. and A.A.) independently extracted the data, using a pre-defined standardized data extraction form. Data extraction included: study characteristics (author, journal, year of publication, study design, study duration), study population (total number of patients and number of patients overexpressing HER2, when applicable), CTRCT definition, CTRCT events, investigated genetic and molecular determinants, methods of investigations and results.

Furthermore, patients’ characteristics (e.g., age) and follow-up were exported if available. Microsoft Office Excel was used for data extraction.

### Risk of bias assessment, quality, and validity of included studies

The risk of bias and quality of the included studies were assessed by the two independent reviewers (M.L. and A.A.) including the use of Newcastle-Ottawa Scale [22] (Table 2). All relevant discrepancies were resolved by discussion until consensus achieved between the two reviewers. The quality score rating was determined for each publication on the Newcastle-Ottawa Scale, with ≥8/9 stars representing observational studies of higher quality.

**Table 2:** Risk of bias assessment of studies included in the in the systematic review

Meta-data			Methodology	Newcastle-Ottawa Scale			
Author	Publication Date	Origin	Study design*	*Selection*	*Comparability*	*Exposure*	*Quality*
**Gómez Peña et al** [15]	2015	Spain	+−	****	*	***	8
**Beauclair et al** [16]	2007	France	+−	****	*	***	8
**Lemieux et al** [17]	2013	Canada	–	****	**	***	9
**Roca L et al** [2]	2013	France	++	****	*	***	8
**Tan et al** [18]	2020	China	+−	****	*	***	8
**Peddi et al** [19]	2022	United States	−+	****	**	***	9
**Stanton et al** [20]	2015	United States	–	****	*	***	8
**Boekhout et al** [21]	2016	Korea	++	****	**	***	9
**Serie et al** [23]	2017	United States	−+	****	**	***	9
**Udagawa et al** [24]	2018	Japan	–	****	**	***	9
**Nakano et al** [25]	2019	Japan	–	****	**	***	9
**Wang et al** [26]	2019	United States	–	**	**	**	6
**Zhang et al** [27]	2020	China	+−	****	**	***	9
**Feng et al** [28]	2021	China	–	****	**	**	8
Mean Newcastle-Ottawa Scale score							8.3

*Abbreviations*: *NA* Not Available, *NR* Not Reported; *Study design: Prospective (+), Retrospective (−); single centre (−), multicentre (+)

Maximum quality score = 9; 0–7 points were considered lower quality, and 8–9 points were considered as higher quality

## Results

**Fig. 1:**
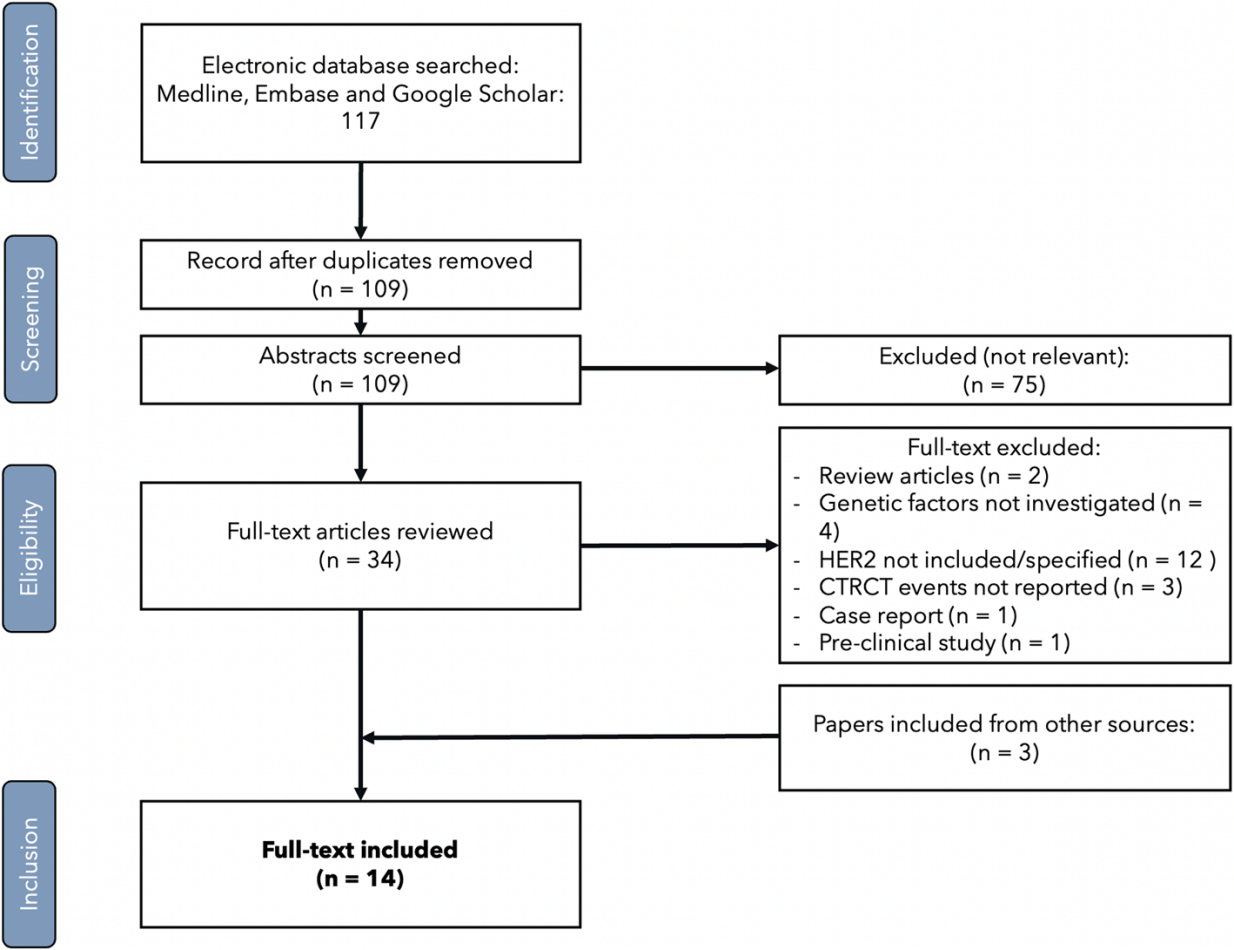
PRISMA flow chart reporting the studies selection process

The literature search retrieved 117 studies. After the removal of duplicates, 109 studies remained. After the revision of all abstracts, another 75 studies were excluded due to irrelevance. The remaining 34 articles underwent full text review and 11 met the predefined inclusion criteria. Three more studies were included from the bibliography scanning making a total of 14 papers included for the present review (Fig. 1). Overall, this systematic review included 3108 patients, among 14 studies. Details of the included studies are reported in Table 3.

**Table 3:** Metadata of the 13 studies included in the systematic review

Author	Date of Publication	Journal	Database	Study design	No of patients	Study Duration (months)
Gómez Peña et al. [15]	2015	Pharmacogenetics and Genomics	Medline	CCS	78	12
Beauclair et al. [16]	2007	Annals of Oncology	Medline	CCS	61	22.4
Lemieux et al. [17]	2013	Anticancer Research	Medline	CCS	73	NA
Roca L et al. [2]	2013	Breast Cancer Research and Treatment	Medline	CCS	132	46.8
Tan et al. [18]	2020	International Journal of Clinical and Experimental Pathology	Medline	CCS	91	15
Peddi et al. [19]	2022	Clinical Cancer Research	Medline	RCT	662	120
Stanton et al. [20]	2015	BMC Cancer	Medline	CCS	140	NA
Boekhout et al. [21]	2016	JAMA oncology	Medline	CCS	206	21
Serie et al. [23]	2017	Journal of cardiovascular development and disease	Medline	Post-hoc analysis	800	72
Udagawa et al. [24]	2018	Cancer science	Medline	CCS	243	NA
Nakano et al. [25]	2019	Biological and pharmaceutical bulletin	Medline	CS	481	37.7
Wang et al. [26]	2019	Medicine (Baltimore)	Medline	Pilot study	3	NA
Zhang et al. [27]	2020	Frontiers in oncology	Medline	CCS	65	NA
Feng et al. [28]	2021	J Breast Cancer	Mediline	CCS	72	15

*Abbreviations*: *NA* Not Available, *RCT* Randomized control trial, *CS* Cohort study, *CCS* Cross sectional, *IV* In-vitro

### Genetic and molecular markers of cardiotoxicity

Three groups of markers associated with CTRCT were identified:

1. HER2-related SNPs
2. Non HER2-related SNPs
3. RNA-related molecular markers

### HER2-related SNPs

A current review of the Exome Variant Server (http://evs.gs.washington.edu/EVS) for ERBB2 retrieved 238 missense SNPs [29]. Despite the multitude of SNPs, hitherto only 2 have been associated with CTRCT: HER2 655 Ile/Val and HER2 1170 Pro/Ala.

### HER2 655 Ile/Val polymorphism

This HER2-related SNP consists of the nucleobase change from Adenine to Guanine (A > G), translating into amino acid change from Isoleucine to Valine (Ile/Val).

Five observational studies [2, 15–18] and one RCT [19] comprising 1097 patients reported the relationship between HER2 655 Ile/Val SNP and the occurrence of CTRCT (details in Table 1). Distribution of HER2 655 Ile/Val SNP was available for every study.

Overall, HER2 655 Ile/Val SNP was present in 392 (35.7%) patients with HER2 positive BC who underwent targeted therapy. A total of 184 (16.8%) women suffered from CTRCT. The heterozygous genotype AG (Ile/Val carriers) was associated with CTRCT (Odds Ratio (OR) range from 3.4 to 8.0, all *p* < 0.05) in all studies but one (Table 1).

**Fig. 2:**
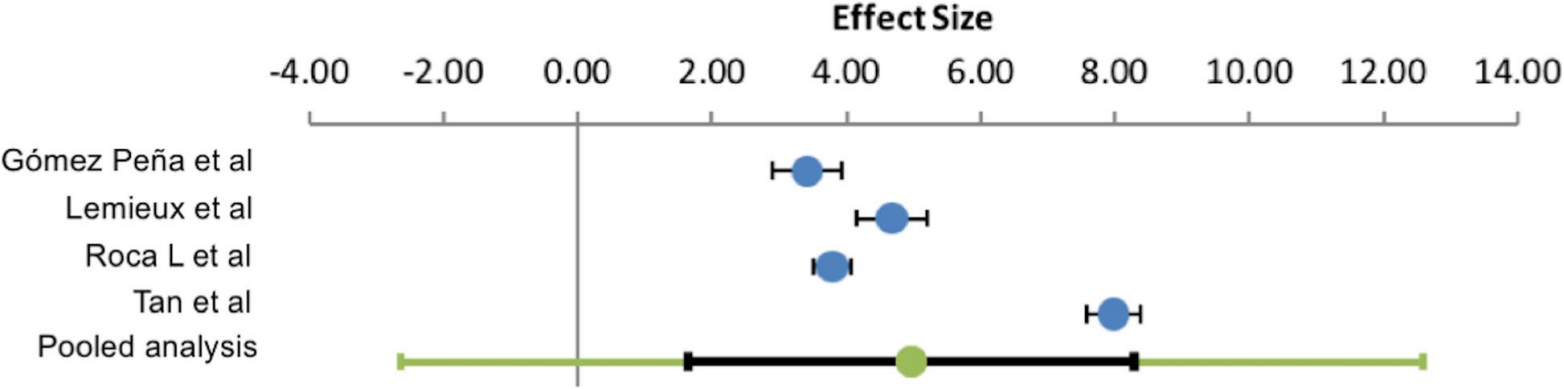
Pooled analysis of HER2 655 SNP association with CTRCT

The combined OR of the 4 studies presenting mergeable data amounted to 4.97 (95% CI 1.65–8.29) (Fig. 2).

### HER2 1170 pro/ala polymorphism

This HER2-related SNP consists of the nucleobase change from Cytosine to Guanine (C > G), translating into amino acid change from Proline to Alanine (Pro/Ala).

Two studies [20, 21], including a total of 346 patients (Table 1), reported a significant association between HER2 1170 Pro/Ala SNP and CTRCT. Genotype details were available only for one study [20] reporting the presence of the SNP/variant in 111(79.3%) patients (both in the heterozygous [CG] and homozygous [GG] form). Cardiotoxicity occurred in 65(18.8%) patients.

Both studies found the presence of the SNP as a protective factor against CTRCT. Specifically, Stanton et al. [20] demonstrated the CC genotype (Pro/Pro carriers) was independently associated with CTRCT (OR 2.60, *p* = 0.046) as compared to SNP carriers C/G (Pro/Ala) and G/G (Ala/Ala) variants. Similarly, Boekhout et al. [21] reported that the homozygous genotype variant G/G (Ala/Ala carriers) was associated with a lower likelihood of cardiac events (OR 0.09, 95% CI 0.02–0.45, *p* = 0.003).

### Non HER2-related SNPs

A consistent group of novel SNPs have been identified as potential markers of CTRCT using GWAS. These SNPs do not directly affect HER2 signalling, rather they are linked to known cardiomyopathy genes or to genes regulating cardiomyocytes apoptosis and metabolism.

Serie et al., analysed the association of genetic variants with CTRCT, across 72 known cardiomyopathy genes, in 800 patients who were treated with doxorubicin and trastuzumab from the N9831 clinical trial. The incidence of cardiotoxicity was 37.3% over 6 years. Genes VCL, DMD, OBSCN, RYR2, TPM1, KCNQ1, JAG1, SGCD, SCN5A, RBM20, SCN4B, TTN and CACNA1C (13/72) showed at least one SNP with evidence of association with chemotherapy and trastuzumab induced decline in LVEF (*p* < 0.05). The most significant association was a DMD intronic variant, rs12559939, *p* = 0.0005 [23] (Supplementary Table 2).

In a retrospective study by Udagawa et al. [24], CTRCT was seen in 19(7.8%) of 243 patients following trastuzumab therapy, of whom 175 had primary site breast cancer. They discovered a total of 239,360 genetic variants in 9 out of the 19 CTRCT cases. The strongest association with CTRCT was found for a locus on chromosome 6q12 (rs139944387, gene EYS, p-combined min = 0.00056, OR = 13.73, 95% Confidence Interval (CI) 4.27–44.21).

A more recent case-control study by Nakano et al. [25], reported cardiotoxicity in 6.2% (24/387) patients treated with trastuzumab. 9 SNPs were identified in five loci associated with CTRCT: rs9316695, rs9527156, rs12583122 and rs11617903 on chromosome 13q14.3, (p-combined = 6.00 × 10–6, 8.64 × 10–6, 1.92 × 10–5 and 2.22 × 10–5, respectively), rs1383149 and rs12372962 on chromosome 15q26.3 (*p*-combined = 1.01 × 10–4 and 1.15 × 10–4, respectively), rs7406710 on chromosome 17q25.3, (p-combined = 1.07 × 10–4), rs11932853 on chromosome 4q25 (p-combined = 1.42 × 10–4), and rs8032978 on chromosome 15q26.3 (p-combined = 1.60 × 10–4). The last one also showed a strong association with CTRCT in patients with hormone receptor positive breast cancer (*p* = 6.55 × 10–5, OR = 8.53, 95% CI = 2.91–23.39).

To the best of our knowledge, in addition to SNPs, no other genetic mutations (i.e., point mutations other than SNPs, insertions, deletions) have been found associated with CTRCT in HER2-positive BC. In the two studies conducted by Serie at al. and Udagawa et al. [23, 24], the authors investigated any genetic variant possibly linked to trastuzumab CTRCT, however, only SNPs were significantly associated with cardiotoxicity.

In the future, new identified cardiomyopathies related genetic variants (i.e., novel desmin (DES) indel mutation [30]) could represent additional research targets also in the Cardio-Oncology field.

### RNA-related molecular markers

#### RNA sequencing markers

The association between trastuzumab-induced CTRCT and the altered expression profile (i.e., upregulation or downregulation) of several genes, using RNA sequencing, has also been reported. Wang et al. compared the single cancer cell expression profile between trastuzumab-treated and nontreated HER2+ breast cancer patients. They identified a 48-gene signature (genetic expression alteration) related to cell death of cardiomyocytes. Among these 48 genes, 14 genes were upregulated, and 34 genes were downregulated by trastuzumab treatment [26] (Supplementary Table 3).

### Micro ribonucleic acids (miRNAs)

Little evidence is available about miRNAs and their association with CTRCT in HER2-positive BC patients. Zhang S et al., investigated the role of miRNA-222-3p in a prospective observational study including 65 patients receiving neoadjuvant therapy from two clinical trials (registered as SHPD001 (NCT02199418) and SHPD002 (NCT02221999)). The incidence of cardiotoxicity was 55.3% (36/65). They reported that the overexpression of serum miRNA-222-3p was an independent protective factor against absolute (OR = 0.410, 95% CI: 0.175–0.962, *p* = 0.040), and relative (OR = 0.394, 95% CI: 0.166–0.937, *p* = 0.035) LVEF reduction, respectively) [27] (Supplementary Table 4). Pre-clinical studies revealed miRNA-222-3p could upregulate HER2 signalling pathway in fulvestrant-resistant breast cancer cells and inhibit the autophagy of cardiac myocytes in mice [31]. Indeed, it is noteworthy that they also reported that higher level of serum miRNA-222-3p was associated with poor oncologic outcome manifested as an inferior pathologic complete response (pCR) rate to neoadjuvant therapy, which might be attributed to trastuzumab-resistance.

Another study [28], including 72 HER2 BC patients undergoing adjuvant chemotherapy with epirubicin/cyclophosphamide followed by docetaxel plus trastuzumab, was conducted to investigate the association of miRNA-130a and CTRCT. CTRCT was identified in 17% of cases. There was an increase in miRNA-130a expression during treatment in all patients, however this increase was greater in those who developed CTRCT. Interestingly, the authors found that miRNA-130a could accurately distinguish CTRCT patients from non-CTRCT ones (AUC = 0.783; 95% CI = 0.647–0.920).

### Other RNA related molecular markers

RNA-related molecular markers include additional molecules, such as long non-coding RNA. Although current evidence of direct association between such molecules and CTRCT in HER2-positive BC is not available, several studies investigating their role in cancer progression/diagnosis may offer interesting input for future dedicated research.

Myocardial infarction associated transcript (MIAT) is a long non-coding RNA that have key-role in several diseases, including myocardial infarction [32]. In a recent study, MIAT expression was found upregulated in.

ER, PR, HER2-positive BC tissues, and its downregulation promoted apoptosis and significantly decreased migration of BC cells. Although no investigations have been conducted to explore its eventual role in HER2-positive BC CTRCT, these findings suggest its potential simultaneous negative effect in BC progression and risk of myocardial infarction in these patients [33].

Other indirect evidence suggests a potential role of the long non-coding RNA HOX transcript antisense RNA.

(HOTAIR) in CTRCT [34]. Indeed, while on one side when downregulated it increases Trastuzumab cancer cells sensitivity [35], on the other side, its downregulation reduces the protection of cardio myocytes to ischemia-reperfusion injury. If these two effects may combine to increase CTRCT during Trastuzumab treatment deserves further investigations [36].

Other RNA molecules (i.e., circular RNA CircITCH [37]) have been associated with doxorubicin CTRCT in pre-clinical experiments, however related studies did not specifically address breast cancer tissue, and the current evidence is too limited to hypothesize a role in HER2-positive BC.

### Quality assessment of included studies

The NOS scores of the included 13 studies included in this systematic review ranged from six to nine with median NOS of eight. Eleven studies are considered of higher quality, and one study of lower quality.

## Discussion

**Fig. 3:**
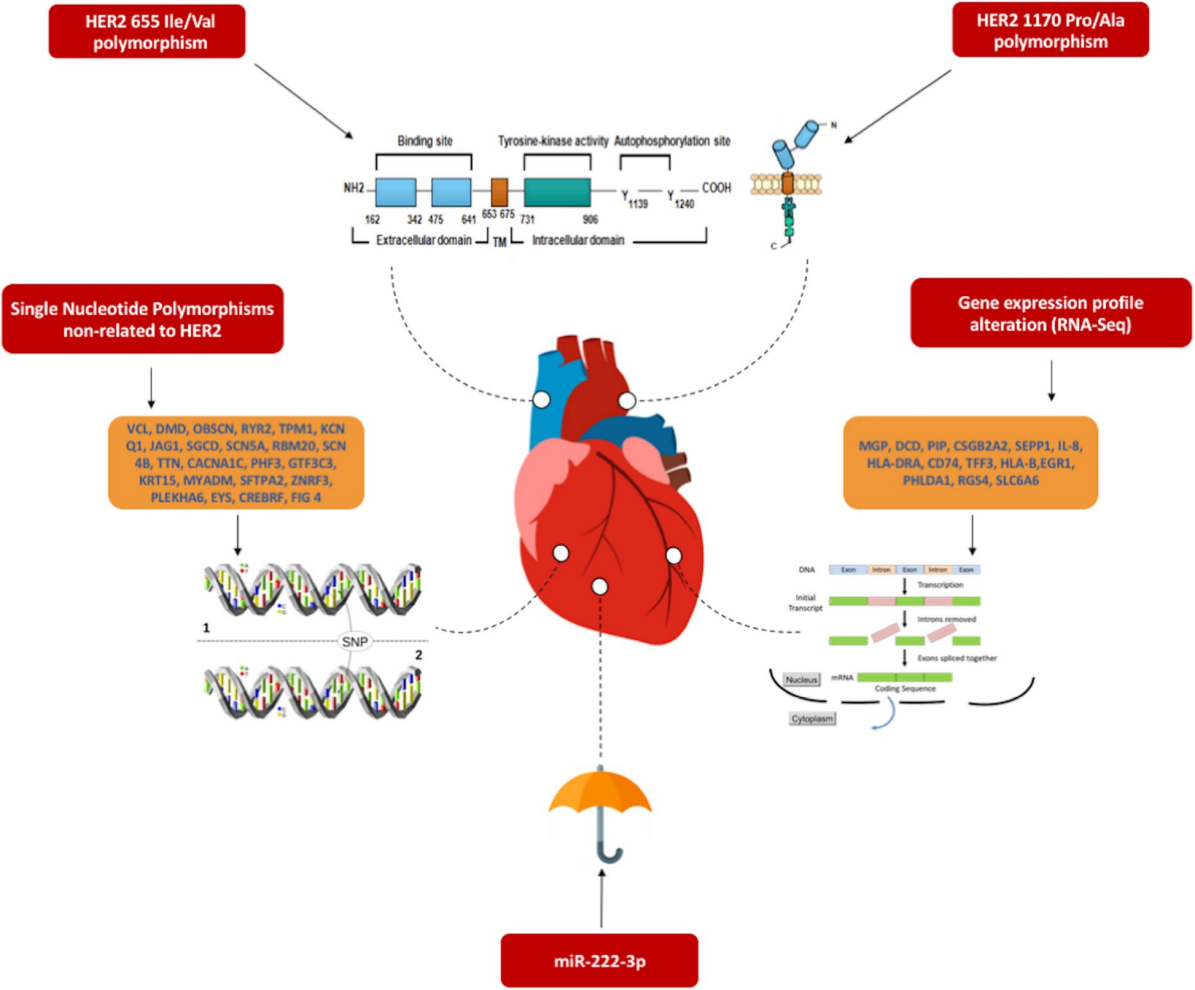
Overview of the genetic determinants of CTRCT in HER2+ breast cancer patients

To the best of our knowledge, this is the first systematic review synthetizing the evidence regarding the genetic and molecular markers of CTRCT in HER2 positive breast cancer patients. The main findings of the present study are: 1) multiple SNPs including HER2-related and HER2 non-related are identified as potential genetic markers of CTRCT in HER2 positive patients with breast cancer; 2) evidence regarding HER-2 related genetic markers is limited and somewhat contradictory 3) there is emerging evidence from gene expression profiling of mRNA markers associated with CTRCT; and 4) miRNAs represent an attractive research field for future CTRCT marker development (Fig. 3). However, supporting evidence of genetic markers of CTRCT to date derives from monocentric and/or small series, warranting further research to confirm their clinical application as risk predictors.

### Evidence and limitations of HER2-related SNPs and trastuzumab-induced cardiotoxicity

**Fig. 4:**
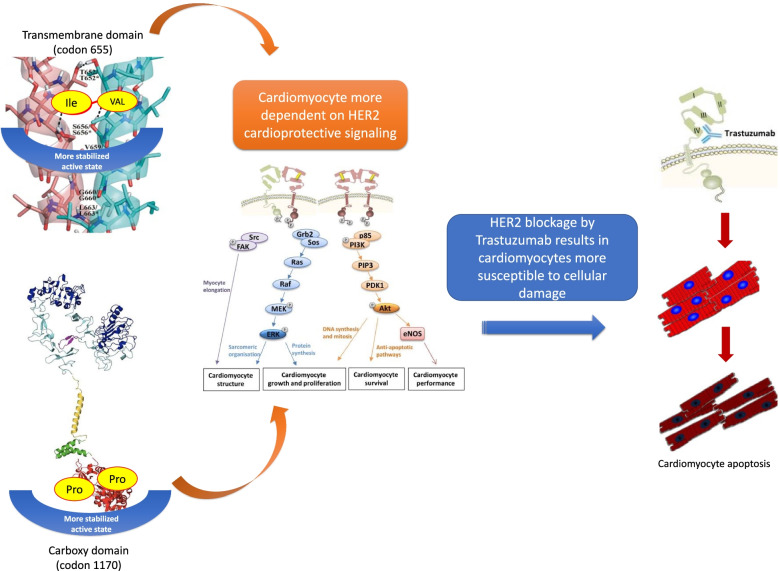
HER2 related potential CTRCT mechanisms. The HER2 protein is described as a homodimer from molecular studies. When the Valine amino acid substitutes Isoleucine (HER2 655 Ile/Val SNP) in the transmembrane domain (top left), the HER2 protein assumes a heterozygous configuration, resulting in an excessively stabilized active state [28, 31]. In the presence of HER2 1170 Pro/Ala SNP, the proline amino acid in the carboxy domain (bottom left) has a secondary amide structure that allow more stable hydrogen binding of nearby amino acids [40]. Both changes lead cardiomyocytes to be especially dependent to HER2 signalling, responsible for their growth, survival and performance. In these cases, the blockage of the overactivated HER2 by monoclonal antibodies like trastuzumab, critically reduce the HER2 protective role of cardiomyocytes, making them more susceptible to cellular damage and secondary function alteration

Although different mechanisms of CTRCT have been hypothesized [38], HER2 signalling represents a central element for cardiomyopathy development in patients overexpressing HER2 [39]. Even if trastuzumab induced cardiotoxicity is often reversible (type II effect, without cell destruction), its long-term effects are unclear, and it often leads to trastuzumab treatment suspension with consequently negative impact on the oncologic prognosis. This laid the foundation for several investigations on HER2 alterations including SNPs, potentially responsible for increased trastuzumab sensitivity, resulting in a downstream modified effect on cardiomyocyte function (Fig. 4).

A SNP is a variation at a single base pair position in a DNA sequence among individuals, defined as present in more than 1% of a population [41]. SNPs are not just associated with genes but can also occur in noncoding regions of DNA. Although a particular SNP may not cause a disorder or phenotypic effect, some SNPs are associated with certain diseases or might influence therapy efficacy or toxicity.

Two HER2-related SNPs were found to be associated with CTRCT in the present review: HER2 655 Ile/Val SNP, codifying for the transmembrane domain; and HER2 1170 Pro/Ala SNP encoding for the carboxy terminus of the HER2 protein.

HER2 655 Ile/Val SNP, leading to an amino acid change from Isoleucine into Valine was an independent predictor of CTRCT in most of the studies (Ile/Val phenotype). This was confirmed by a meta-analysis including 344 patients, of whom 43 suffered from CTRCT. Of these, 29 (67%) patients did present the Ile/Val phenotype while 14 (33%) patients did not (Ile/Ile), resulting in an OR for cardiotoxicity of 5.35 (95% CI 2.55–11.73, *p* < 0.0001) [15]. Although these findings corroborate the hypotheses that the HER2 signalling pathway, when altered from HER2 target therapy, plays a relevant role in CTRCT, a larger retrospective analysis of a randomized trial reported opposite results. Peddi et al. [19], indeed, found no correlation between HER2 655 Ile/Val SNP and CTRCT.

Interestingly, in all but two of these studies [2, 15–17], no CTRCT events were reported among the Val/Val homozygous patients (G/G genotype). Although it can be explained by the very low (3.9%) incidence of the Val/Val phenotype, reducing the possibility of cardiotoxicity, these finding sheds light on the potential HER2-related mechanism implicated in cardiac injury. The presence of the Valine amino acid, per se is not sufficient to lead to cardiac effects. In contrast, the heterozygous combination (Ile/Val) seems more likely involved in cardiotoxicity. These data support the assumption derives from molecular studies, which described the HER2 protein as a homodimer. Therefore, in the presence of the heterozygous genotype it assumes an excessively stabilized active state, rendering the cardiomyocytes more dependent upon HER2-signaling and therefore highly sensitive to trastuzumab [42, 43].

On the other hand, limited evidence is available regarding trastuzumab-induced cardiotoxicity and the SNP related to codon 1170 of HER2 protein, leading amino acid change from Proline to Alanine (Pro/Ala). Two studies reported an association between the variant form (Alanine carriers) and the risk of CTRCT. Interestingly, this SNP was found to be a protective factor associated with fewer cardiotoxicity events [20, 21]. The underlining protective mechanism of the Alanine presence is still unclear. The HER2 protein modification induced by this SNP does not concern the trastuzumab or the ligand binding sites, but the carboxy domain containing tyrosine residues representing the phosphorylation sites for the kinase. Although proline and alanine both present non-polar side chains, proline has a secondary amide structure that may allow more stable hydrogen binding of nearby amino acids [40]. However, the role of altered downstream signalling and CTRCT is yet to be established.

In contrast to the studies investigating non-HER2 SNPs or RNA-related markers, some of these analysed also the potential relationship between HER2 SNPs and known cardiotoxicity clinical risk factors, such as hypertension, in increasing the trastuzumab-related CTRCT.

Specifically, hypertension was not found associated to HER2 1170 Pro/Ala SNP in predicting CTRCT [44]. In regards to HER2 655 Ile/Val SNP, while one study did not show any association with baseline hypertension and CTRCT [18], Lemieux et al. reported an increased odd of CTRCT in patients also presenting hypertension. Nevertheless, this did not result a significant predictor at the multivariate analysis (adjusted OR 1.81, 95%CI 0.78–4.21, p 0.17), suggesting no link with HER2 SNPs related CTRCT. If other genetic or RNA markers present associations with clinical risk factors is still undetermined and warrants further investigations.

Although the consistency of the data among most of studies, the association of these SNPs with CTRCT is not yet definitively established. Firstly, the evidence supporting their association is based on relatively small sample studies. Furthermore, no significant association between trastuzumab-induced reduction in LVEF and the above mentioned HER2-related SNPs, HER2 655 Ile/Val (OR 0.941, 95% CI 0.71–1.25, *p* = 0.67), and the HER2 1170 Pro/Ala (OR 1.034, 95% CI 0.80–1.34, *p* = 0.80) was observed in the largest study to date [45],

Of note, these studies are limited by retrospective design, lack of standardized protocols of imaging acquisition and analysis. Furthermore, different definitions for cardiotoxicity were used among different studies. Therefore, a definitive conclusion on the HER2-related SNPs is yet to be established.

### Non HER2-related SNPs and trastuzumab-induced cardiotoxicity

Studies investigating the presence of genetic variants in known genes related to cardiomyopathies, found novel SNPs unrelated to HER2, potentially associated with CTRCT in HER2 BC patients.

The group of Serie DJ et al., tried to find putative modifying variants across 72 known genes causing cardiomyopathies. They reported the strongest association with obscurin gene which was identified as causative of dilated cardiomyopathy (DCM) [46]. They suggest that common and rare variants in obscurin gene may contribute to DCM burden or perhaps modify disease progression or outcome.

Another study found a strong association between a locus on chromosome 6q12 and trastuzumab-induced cardiotoxicity (rs139944387 in Eyes Shut Homolog (EYS)).

EYS gene - is a Protein Coding gene – which is mutated in autosomal recessive retinitis pigmentosa. The product of this gene contains multiple epidermal growth factor (EGF)-like and LamG domains. EYS might affect the efficiency of HER2 signal transduction in cardiac myocytes, and interindividual difference of EYS function caused by the genetic variant(s) might affect the incidence of trastuzumab-induced cardiotoxicity [24].

Another potential association was found in the Japanese population, where Nakano et al. reported that locus rs8032978 on chromosome 15q26.3 might be a promising marker of trastuzumab-induced cardiotoxicity in patients with hormone receptor positive breast cancer. Locus rs8032978 on chromosome 15q26.3, was located 44 kb downstream from the PCSK6 gene. PCSK6 (Proprotein convertase subtilisins/kexins) activate Corin which is a type II transmembrane serine protease found mainly in the heart. It plays an essential role in the regulation of water and salt balance by converting natriuretic peptides to their active form, consequently, promoting vasodilation, diuresis and natriuresis, and prevent cardiac remodelling in heart failure patients. Therefore, impaired activity and/or gene expression of PCSK6 might contribute to the inability to recover the cardiac function in damaged heart due to insufficient activation of natriuretic peptides [47].

Interestingly, they also identified five SNPs, (rs9316695, rs28415722, rs7406710, rs11932853 and rs8032978), as independent predictors of trastuzumab-induced cardiotoxicity (*p* = 2.82 × 10^− 4^ - 4.15 × 10^− 3^) and investigated the combined effects of these five loci on the risk of cardiotoxicity in patients treated with trastuzumab by using a scoring system. The author assigned a score of 2 to individuals homozygous for risk allele, 1 to individuals heterozygous for risk allele, and 0 to individuals homozygous for non-risk allele at rs9316695, rs11932853 and rs8032978, respectively. A score of 2 to individuals homozygous for the risk allele and 0 to individuals with the other genotypes was assigned at rs28415722 and rs7406710. The final individual score derived from the sum of the scores for each SNP. The proportion of patients with trastuzumab-induced cardiotoxicity was likely to be increased in groups with higher prediction scores; the incidences of the cardiotoxicity were 1.8% (8/441) in the score 0–4 group, 36.4% (8/22) in the score 5 group, 22.2% (2/9) in the score 6 group, 75.0% (3/4) in the score 7 group and 80.0% (4/5) in the score 8 group.

This score system to our knowledge is the first one to predict the risk of cardiotoxicity [25].

### RNA sequencing-related markers and trastuzumab-induced cardiotoxicity

RNA-sequencing has multiple applications in breast cancer research including exploring tumour heterogeneity, analysis of cell-cell communications, regulatory single-cell states and immune cell distributions [48].

This technique also allowed the detection of several genes, with dysregulated expression profile induced by trastuzumab treatment. Interestingly, some of the upregulated genes such as SPP1 and HMOX1 are known to increase the apoptosis of cardiac myocytes. Also, many of the downregulated genes, such as TIMP1 and NAMPT, are known to decrease apoptosis of cardiomyocytes. CXADR one of the downregulated genes, are essential for cardiomyocyte development. The dysregulation of these genes could increase apoptosis of cardiomyocytes and disrupt the cardiomyocytes development, which could explain trastuzumab-mediated cardiotoxicity [26].

Similarly, Necela et al. [44] investigated in a pre-clinical study the whole genetic expression profile of human induced pluripotent stem cell-derived cardiomyocytes, treated with trastuzumab and lapatinib vs those untreated. They identified a total of 38 genes, which expression was significantly altered. Some of them (EGR1, PHLDA1, RGS4 and SLC6A6 genes) are known to be related with cardiac dysfunction and ischemic injury. EGR1 and PHLDA1 that resulted down-regulated during trastuzumab treatment, are significantly upregulated in ischemic preconditioning suggesting that their expression may help against CTRCT.

RGS4 gene was also significantly downregulated. Absence of RGS4 gene induces atrial fibrillation and its activation leads to cardioprotective effects due to increased expression of natriuretic peptides in the heart. Likewise, SLC6A6 gene was also significantly downregulated. SLC6A6 is implemented in calcium handling and in protection against ischemia–reperfusion injury, heart failure ischemic heart disease, and diabetic cardiomyopathy.

Identifying early changes in gene expression, mediated by trastuzumab, might represent potential biomarkers of drug-induced cardiotoxicity, thereby facilitating prevention or early intervention to improve outcomes.

### MiRNAs

Most miRNA-oriented studies aimed to identify disease severity and aggressivity without investigating drugs cardiotoxic effect in HER2 positive breast cancer patients [49]. Also, some studies tried to identify miRNAs that can predict responsiveness to treatment with trastuzumab in HER2+ BC patients, the following miRNAs (miR-940, miR-451a, miR-16-5p, and miR-17-3p) were tested by Li et al. and they discovered that the serum-based 4-miRNA signature can effectively distinguish HER2+ BC patients who are sensitive to trastuzumab from the resistant ones [50].

One of the two studies investigating the role of miRNAs in predicting trastuzumab induced cardiotoxicity in HER2+ BC patients, revealed that serum miRNA-222-3p may play an endocrine role protecting the cardiomyocytes. Indeed, it is necessary for cardiomyocyte growth induced by exercise and to protect against adverse cardiac remodelling after ischemic injury [51]. Hence, miR-222-3p overexpression might prevent the heart from trastuzumab-induced injury, and its monitoring may play an important role in CTRCT prevention. However, it is described as “a double-edged sword” since high expression of miR-222-3pin the serum is associated with poor oncologic outcome due to trastuzumab resistance [27].

MiRNA-130a was, on the contrary, found to be a predictor of CTRCT [28]. It is normally expressed in cardiac tissue, and its upregulation in mouse models leads to delayed heart development with thinner ventricular muscle; while the inhibition protects cardiac myocytes from hypoxia-triggered apoptosis. This underlies the basis of its potential involvement in cardiac myopathies, including CTRCT. Interestingly, its inclusion in CTRCT predictive risk models [28] improved their accuracy, allowing a better distinction between patients developing CTRCT and those not.

In addition to the expression of circulating miRNAs, another promising research field regards the genetic variants (SNPs) of miRNAs. Even if not focused on HER2+ breast cancers, pharmacogenomic studies have shown that SNPs causing alterations in the binding site of some miRNAs have a role in CTRCT. For instance, rs3732360 and rs3732359 are 2 SNPs that have been demonstrated to alter the binding site for 2 miRNAs (miR 500a-3p, miR 532-3p) that are known to play a role in breast cancer doxorubicin induced CTRCT [52]. As consequence, the detection of these genetic variants may help to estimate the increased or reduced risk of CTRCT in a patient.

Lastly, we do have to mention that some miRNAs are dysregulated in BC itself, regardless trastuzumab treatment. Some of them (i.e miR126 [53]) may influence the cardiac function, leading to cardiac events like heart failure, therefore possibly representing a confounder when investigating CTCRT.

## Conclusions and future perspectives

Data on genetic and molecular markers with a predictive or protective role for trastuzumab-induced cardiotoxicity are as of yet limited but encouraging. In particular, although studies addressing genetic variants other than HER2 sound innovatory, at present the supporting evidence is too little to make any clinical inference. Future adequately powered, prospective studies with a focus on the development and validation of highly predictive genetic biomarkers of cardiotoxicity are warranted. Indeed, such studies have the potential to significantly inform clinical practice through prediction or early identification of patients at risk of CTRCT.

On the one side, the detection of genetic variants associated with CTRCT before the initiation of a certain chemotherapy regimen, could help to 1) enhance the patient’s cardiac monitoring to earlier diagnose eventual cardiac dysfunction, 2) promote upfront cardiac protective therapies and 3) target the oncological treatment to avoid, when possible, the most cardiotoxic molecules. Similarly, the identification of protective markers might support more aggressive oncological therapies, if deemed appropriate improve the patient prognosis.

On the other side, the understanding of new mechanisms of CTRCT, for instance through the identification of early changes in genetic expression (i.e. upregulation of apoptotic SPP1 and HMOX1 genes), may represent additional opportunities for targeted therapies, aiming to restore the normal genes expression and consequently the cardiac function.

All together, these strategies have the main goal to allow the patients to pursue an effective/optimal oncological therapy without being harmed by deleterious cardiac effects or the consequences of treatment discontinuation.

## Supplementary Information

**Additional file 1: Supplementary Table 1.** Criteria used for the literature research across Embase, Medline and Google Scholar. **Supplementary Table 2.** Summary of CTRCT events, and related genetic variants in studies investigating genes other than HER2. **Supplementary Table 3.** Top 5 most significantly upregulated and downregulated genes between trastuzumab treated versus nontreated single cancer cells. **Supplementary Table 4.** Association between serum miR-222-3p and cardiotoxicity.

## Data Availability

Not applicable.

## Abbreviations

CI: Confidence interval
CTRCT: Cancer therapy-related cardiotoxicity
DFS: Disease-free survival
EYS: Eyes Shut Homolog
GWAS: Genome-wide association study
HER2: Human epidermal growth factor receptor 2
LVEF: Left ventricular ejection fraction
MAF: Minor allele frequency
MiRNAs: Micro Ribonucleic acids
NAT: Neoadjuvant therapy
OR: Odds ratio
RCTs: Randomized controlled trials
RNA-seq: RNA sequencing
scRNA-Seq: Single cell Ribonucleic acid sequencing
SNPs: Single nucleotide polymorphisms
