# Supplementary material for: Genetic and RNA-related molecular markers of trastuzumab-chemotherapy-associated cardiotoxicity in HER2 positive breast cancer: a systematic review
Source: BMC Cancer. 2022 Apr 12;22:396. doi: 10.1186/s12885-022-09437-z (PMC9004047; doi:10.1186/s12885-022-09437-z)
Supplement: Supplementary file 1 — Additional file 1: Supplementary Table 1. Criteria used for the literature research across Embase, Medline and Google Scholar. Supplementary Table 2. Summary of CTRCT events, and related genetic variants in studies investigating genes other than HER2. Supplementary Table 3. Top 5 most significantly upregulated and downregulated genes between trastuzumab treated versus nontreated single cancer cells. Supplementary Table 4. Association between serum miR-222-3p and cardiotoxicity. [file 12885_2022_9437_MOESM1_ESM.docx]

**Supplementary Table 1. Criteria used for the literature research across Embase, Medline and Google Scholar.**

| **Population** | (breast cancer[Title]) OR (mammary cancer[Title])) OR (adenocarcinoma breast[Title])) OR (breast tumor[Title])) OR (breast adenocarcinoma[Title]) **AND**  (her2[Title/Abstract]) OR (her-2[Title/Abstract])) OR (her2/neu[Title/Abstract])) OR (her2-neu[Title/Abstract])) OR (her2 neu[Title/Abstract])) OR (Herceptin[Title/Abstract])) OR (erbb-2[Title/Abstract])) OR (erbb2[Title/Abstract])) OR (erbb 2[Title/Abstract])) OR (trastuzumab[Title/Abstract]) **AND** |
| --- | --- |
| **Risk factors** | (non-coding RNA[Title/Abstract]) OR (small RNAs[Title/Abstract])) OR (small RNA[Title/Abstract])) OR (microRNA[Title/Abstract])) OR (piRNA[Title/Abstract])) OR (siRNA[Title/Abstract])) OR (snoRNA[Title/Abstract])) OR (snRNA[Title/Abstract])) OR (exRNA[Title/Abstract])) OR (scaRNA[Title/Abstract])) OR (micro RNA[Title/Abstract])) OR (genetic factors[Title/Abstract])) OR (gene[Title/Abstract])) OR (genes[Title/Abstract])) OR (polymorphism[Title/Abstract])) OR (alleles[Title/Abstract])) OR (genetic variants[Title/Abstract]) OR (long non coding RNA[Title/Abstract]) OR (lncRNA[Title/Abstract])) OR (lnc-RNA[Title/Abstract])) OR (long non coding-RNA[Title/Abstract])) OR (long ncRNAs[Title/Abstract])) OR (Long non-coding RNAs[Title/Abstract])) OR (Long non-coding RNA[Title/Abstract])) OR (long ncRNA[Title/Abstract])) OR (circRNA[Title/Abstract])) OR (circular RNA[Title/Abstract])) OR (ceRNA[Title/Abstract])) OR (competing endogenous RNA[Title/Abstract])) OR (competitive endogenous RNA[Title/Abstract])) OR (point mutation[Title/Abstract])) OR (insertion[Title/Abstract])) OR (deletion[Title/Abstract])) OR (indel[Title/Abstract])) **AND** |
| **Outcomes** | (cardiovascular events[Title/Abstract]) OR (cardiovascular adverse events[Title/Abstract])) OR (major adverse cardiovascular events[Title/Abstract])) OR (myocardial infarction[Title/Abstract])) OR (unstable angina[Title/Abstract])) OR (angina[Title/Abstract])) OR (stroke[Title/Abstract])) OR (transient ischemic attack[Title/Abstract])) OR (heart failure[Title/Abstract])) OR (cardiac death[Title/Abstract])) OR (cardiovascular death[Title/Abstract])) OR (cardiac mortality[Title/Abstract])) OR (peripheral vascular disease[Title/Abstract])) OR (myocardial disfunction[Title/Abstract])) OR (myocardial dysfunction[Title/Abstract])) OR (left ventricular dysfunction[Title/Abstract])) OR (ejection fraction[Title/Abstract])) OR (systolic dysfunction[Title/Abstract])) OR (diastolic dysfunction[Title/Abstract])) OR (coronary artery disease[Title/Abstract])) OR (ischemic heart disease[Title/Abstract])) OR (ischemic heart disease[Title/Abstract])) OR (arrhythmias[Title/Abstract])) OR (arterial hypertension[Title/Abstract])) OR (hypertension[Title/Abstract])) OR (thromboembolic disease[Title/Abstract])) OR (pulmonary embolism[Title/Abstract])) OR (pericardial effusion[Title/Abstract])) OR (pericarditis[Title/Abstract])) OR (pericardial disease[Title/Abstract])) OR (cardiomyopathy[Title/Abstract])) OR (QTc[Title/Abstract])) OR (QT[Title/Abstract])) OR (sudden death[Title/Abstract])) OR (cardiotoxicity[Title/Abstract])) OR (cardiac toxicity[Title/Abstract])) OR (cardiovascular toxicity[Title/Abstract])). |
| **Time** | No limit |
| **Language** | English |

# Supplementary Table 2. Summary of CTRCT events, and related genetic variants in studies investigating genes other than HER2.

| Author | Number of investigated  Genes | No of patients | CTRCT definition | Percentage of CTRCT events | Gene name | SNPs number | SNP ID | MAF/RAF | 95%CI | p value |
| --- | --- | --- | --- | --- | --- | --- | --- | --- | --- | --- |
| Serie DJ et al | 72 | 800 | Maximum LVEF decline (any value below the baseline) | 37.3 % | VCL  DMD  OBSCN  RYR2  TPM1  KCNQ1  JAG1  SGCD  SCN5A  RBM20  SCN4B  TTN  CACNA1C | 105  432  55  273  81  881  18  1851  243  57  8  275  216 | rs12250729  rs76974852  rs111748583  rs140820221  rs1795571  rs331317  rs72626080  rs2050074  rs2050076  rs12559939  rs141927233  rs73623943  rs540662  rs1188697  rs4653546  rs56021350  rs61825286  rs435776  rs61825301  rs4653942  rs3795801  rs287611  rs1188710  rs883748  rs80107454  rs2253083  rs5813188  rs8026502  rs79854225  rs57645645  rs73431508  rs12441488  rs80056995  rs16928363  rs2237868  rs74392867  rs79295543  rs77059665  rs28730663  rs35237966  rs72844252  rs231880  rs71476688  rs34861825  rs12419030  rs12419347  rs3748480  rs6860238  rs11129796  rs9832586  rs7430407  rs6790619  rs7645173  rs9311190  rs11711097  rs7432532  rs7426433  rs7433889  rs6599214  rs6599215  rs6599216  rs6599217  rs6599218  rs7613045  rs63200660  rs6599221  rs7627488  rs7636280  rs7069694  rs2181407  rs17831429  rs955917  rs3813250  rs3820978  rs2366751  rs3829746  rs4894029  rs1560221  rs2163009  rs6712785  rs1001238  rs12693162  rs16866420  rs2562839  rs2562838  rs2562836  rs2562830  rs2742327  rs1484116  rs7559166  rs2291313  rs1009281  rs11832738 | 0.25  0.02  0.02  0.05  0.13  0.47  0.09  0.13  0.13  0.19  0.19  0.24  0.29  0.31  0.19  0.18  0.14  0.50  0.18  0.20  0.18  0.49  0.47  0.47  0.04  0.04  0.13  0.13  0.12  0.13  0.14  0.13  0.05  0.05  0.05  0.05  0.05  0.04  0.03  0.39  0.09  0.35  0.03  0.16  0.31  0.31  0.12  0.05  0.09  0.10  0.11  0.07  0.07  0.07  0.22  0.11  0.11  0.13  0.13  0.13  0.13  0.13  0.13  0.12  0.13  0.07  0.02  0.01  0.41  0.28  0.03  0.17  0.22  0.22  0.22  0.22  0.22  0.23  0.23  0.22  0.23  0.21  0.21  0.20  0.20  0.20  0.20  0.20  0.20  0.15  0.20  0.48  0.30 | 0.84 (0.10 to 1.57)  −3.34 (−5.52 to −1.15)  −3.66 (−6.22 to −1.11)  −1.94 (−3.37 to −0.52)  −0.86 (−1.70 to −0.03)  0.85 (0.21 to 1.49)  1.87 (0.76 to 2.97)  1.28 (0.34 to 2.22)  1.32 (0.37 to 2.27)  1.48 (0.65 to 2.30)  1.45 (0.63 to 2.28)  1.18 (0.44 to 1.93)  1.05 (0.34 to 1.76)  1.02 (0.32 to 1.72)  −1.20 (−2.03 to −0.38)  −1.42 (−2.26 to −0.59)  −1.39 (−2.35 to −0.44)  −1.09 (−1.76 to −0.43)  −1.41 (−2.25 to −0.58)  −1.43 (−2.26 to −0.61)  −1.39 (−2.23 to −0.56)  −1.10 (−1.76 to −0.43)  −1.13 (−1.79 to −0.47)  −1.16 (-1.82 to −0.50)  −2.89 (−4.59 to −1.19) 2.10 (0.55 to 3.65)  1.42 (0.49 to 2.34)  1.42 (0.49 to 2.34)  1.26 (0.28 to 2.24)  1.42 (0.49 to 2.34)  1.31 (0.38 to 2.23)  1.42 (0.49 to 2.34)  −1.94 (−3.41 to −0.46)  −1.84 (−3.30 to −0.37)  −1.84 (−3.30 to −0.37)  −1.92 (−3.39 to −0.45)  −1.94 (−3.41 to −0.46)  −2.29 (−3.90 to −0.69)  −2.71 (−4.62 to −0.80)  −0.94 (−1.61 to −0.26)  −1.57 (−2.71 to −0.42)  −0.70 (−1.39 to −0.01)  2.59 (0.87 to 4.32)  1.38 (0.40 to 2.36)  0.89 (0.17 to 1.61)  0.91 (0.19 to 1.63)  1.52 (0.51 to 2.53)  2.24 (0.71 to 3.78)  −1.44 (−2.67 to −0.20) −1.28 (−2.35 to −0.21) −1.43 (−2.44 to −0.41) −1.44 (−2.70 to −0.17) −1.52 (−2.81 to −0.24) −1.56 (−2.85 to −0.27) −0.85 (−1.64 to −0.07) −1.45 (−2.47 to −0.43) −1.50 (−2.52 to −0.48) −1.13 (−2.10 to −0.17) −1.21 (−2.17 to −0.25) −1.27 (−2.23 to −0.31) −1.21 (−2.17 to −0.26) −1.20 (−2.15 to −0.25) −1.20 (−2.15 to −0.24) −1.36 (−2.33 to −0.40) −1.19 (−2.14 to −0.25) −1.30 (−2.59 to −0.01) −2.99 (−5.55 to −0.43) −2.96 (−5.68 to −0.23)  −0.79 (−1.45 to −0.13) −0.76 (−1.47 to −0.06) −2.68 (−4.58 to −0.78)  1.07 (0.22 to 1.93)  0.85 (0.06 to 1.63)  0.79 (0.01 to 1.58)  0.81 (0.03 to 1.60)  0.81 (0.03 to 1.60)  0.81 (0.02 to 1.59)  0.82 (0.04 to 1.60)  0.82 (0.04 to 1.61)  0.82 (0.03 to 1.60)  0.82 (0.04 to 1.61)  0.86 (0.06 to 1.65)  0.82 (0.02 to 1.60)  0.83 (0.01 to 1.64)  0.83 (0.02 to 1.64)  0.83 (0.01 to 1.64)  0.82 (0.01 to 1.63)  0.82 (0.02 to 1.63)  0.88 (0.08 to 1.68)  1.08 (0.19 to 1.98)  0.86 (0.06 to 1.67)  −0.76 (−1.41 to −0.12) −0.81 (−1.49 to −0.12) | 0.0259  0.0029  0.0051  0.0078  0.0438  0.0090  0.0010  0.0077  0.0065  0.0005  0.0006  0.0020  0.0038  0.0045  0.0044  0.0009  0.0044  0.0014  0.0010  0.0007  0.0011  0.0013  0.0008  0.0006  0.0009  0.0081  0.0027  0.0027  0.0121  0.0027  0.0058  0.0027  0.0102  0.0142  0.0142  0.0106  0.0102  0.0051  0.0056  0.0065  0.0074  0.0466  0.0033  0.0061  0.0160  0.0139  0.0033  0.0043  0.0228  0.0190  0.0060  0.0264  0.0206  0.0180  0.0340  0.053  0.0040  0.0213  0.0142  0.0099  0.0126  0.0142  0.0142  0.0059  0.0137  0.0478  0.0224  0.0337  0.0198  0.0342  0.0058  0.0140  0.0349  0.0486  0.0432  0.0432  0.0453  0.0406  0.0399  0.0421  0.0399  0.0344  0.0437  0.0467  0.0449  0.0467  0.0472  0.0460  0.0314  0.0191  0.0366  0.0204  0.0215 |
| Udagawa et al | 10 | 243 | ≥10% decrease of LVEF compared to baseline | 7.8 % | PHF3  GTF3C3  KRT15  MYADM  SFTPA2  ZNRF3  PLEKHA6  EYS  CREBRF  FIG 4 |  | rs139503277  rs146213213  rs78272919  rs140387622  rs150273659  rs5762940  rs149581993  rs139944387  rs201763080  rs56378532 | 0.167  0.167  0.222  0.278  0.167  0.222  0.111  0.167  0.111  0.222 | 41.2 (10.4‐162.7)  26.6 (7.1‐100.5)  13.4 (4.2‐42.2)  10.2 (3.5‐29.2)  20.9 (5.6‐77.5)  12.8 (4.1‐40.1)  99.8 (13.2‐752.6)  19.6 (5.3‐72.5)  68.8 (11.7‐402.5)  10.1 (3.2‐31.5) | .00014  .00042  .00055  .00043  .00080  .00065  .00069  .00091  .00086  .00094 |
| Nakano HM at al |  | 481 | LVEF <45% or LVEF <50% with an absolute decrease of 10% from baseline | 6.2% |  |  | rs9316695  rs28415722  rs7406710  rs11932853  rs8032978 | 0.38  0.62  0.90  0.66  0.18 | 4.46 (2.30–8.47)  5.48 (2.21–13.69)  6.64 (2.19–27.01)  3.20 (1.70–6.23)  5.83 (2.30–13.51) | .00002  .00036  .00041  .00005  .00014 |

**Abbreviations:** NA=Not Available**.**

**Supplementary Table 3: Top 5 most significantly upregulated and downregulated genes between trastuzumab treated versus nontreated single cancer cells.**

| **Top 5 upregulated genes ID** | **MGP** | **DCD** | **PIP** | **SCGB2A2** | **SEPP1** |
| --- | --- | --- | --- | --- | --- |
| **Top 5 downregulated genes ID** | **IL-8** | **HLA-DRA** | **CD74** | **TFF3** | **HLA-B** |

**Supplementary Table 4: Association between serum miR-222-3p and cardiotoxicity:**

| Adverse event | N | OR | 95% CI | P |
| --- | --- | --- | --- | --- |
| aLVEF | **36** | **0.394** | **0.166–0.937** | **0.035** |
| rLVEF | **36** | **0.410** | **0.175–0.962** | **0.040** |

**Abbreviations:** aLVEF absolute drop of left ventricular ejection fraction from baseline, rLVEF relative drop of left ventricular ejection fraction from baseline
